# Significance of CD47 and Its Association With Tumor Immune Microenvironment Heterogeneity in Ovarian Cancer

**DOI:** 10.3389/fimmu.2021.768115

**Published:** 2021-12-13

**Authors:** Lan Yu, Yi Ding, Ting Wan, Ting Deng, He Huang, Jihong Liu

**Affiliations:** ^1^ Department of Gynecologic Oncology, State Key Laboratory of Oncology in South China, Collaborative Innovation Center for Cancer Medicine, Sun Yat-Sen University Cancer Center, Guangzhou, China; ^2^ Department of Obstetrics and Gynecology, The First Affiliated Hospital of Guangzhou Medical University, Guangzhou, China

**Keywords:** CD47, scRNA-seq, tumor microenvironment, heterogeneity, ovarian cancer

## Abstract

**Background:**

It was reported that tumor heterogeneity and the surrounding tumor microenvironment (TME) in ovarian cancer affects immunotherapy efficacy and patient outcomes. And the TME of ovarian cancer is intrinsically heterogeneous. CD47 plays vital roles in cell functional behavior and immune homeostasis relating to cancer prognosis. But how it affects TME and its contribution to heterogeneity in ovarian cancer has not been fully illustrated. Therefore, we aimed to identify a prognostic biomarker which may help explain tumor immune microenvironment heterogeneity of ovarian cancer.

**Methods:**

Cancer single-cell state atlas (CancerSEA) was used to evaluate functional role of CD47. Several bioinformatics database including Oncomine, Gene Expression Profiling Interaction Analysis (GEPIA), Tumor Immune Estimation Resource (TIMER), The Human Protein Atlas (HPA), Ualcan and Kaplan-Meier plotter (KM plotter) were applied to illustrate correlation of CD47 with ovarian cancer prognosis and immune infiltration. Tumor Immune Single-cell Hub (TISCH) single cell database was employed to evaluate correlation of CD47 with tumor microenvironment. GeneMANIA was implemented to identify regulation networks of CD47. Differentially expressed genes (DEGs) between CD47 high and low expression groups were analyzed with R package DESeq2. Kyoto encyclopedia of genes and genomes (KEGG) and Gene Set Enrichment Analysis (GSEA) were utilized to explore how CD47 affect the immune related cell signaling pathway.

**Results:**

CD47 expression was upregulated and connected to worse OS and PFS in ovarian cancer. Close relation was found between CD47 expression level and immune infiltration in ovarian cancer, especially with Treg cells, Monocytes, Macrophages and T cell exhaustion (*P*<0.05). The CD47 expression level was relatively low in plasma cells, dendritic cells and Mono/Macro cells of OV_GSE115007, in myofibroblasts, fibroblasts and endothelial cells of OV_GSE118828, compared to malignant cells of OV_GSE118828 dataset. The cell components and distribution in primary and metastatic ovarian cancer are quite distinct, which may lead to TME heterogeneity of ovarian cancer.

**Conclusion:**

Our results indicated that CD47 is closely correlated to ovarian cancer immune microenvironment and might induce ovarian cancer heterogeneity. Therefore, CD47 may be used as a candidate prognostic biomarker and provide us with new insights into potential immunotherapy in ovarian cancer patients.

## Introduction

Ovarian cancer is one of the most lethal malignancies among women worldwide. Even with bulk surgery and adjuvant chemotherapy, it shows a high fatality rate of 70% in late stage patients (1). Despite advances in maintenance therapy of bevacizumab or PARP inhibitors, most ovarian cancer patients have a high recurrence rate within 5 years of diagnosis (2). It was reported that immunotherapy efficacy and patient outcomes were closely related to the tumor heterogeneity and the surrounding tumor microenvironment (TME) in ovarian cancer (3, 4). Because TME of ovarian cancer patients is intrinsically heterogeneous, successful treatments towards ovarian cancer are challenging (4, 5). Therefore, we aimed to identify a prognostic biomarker which may help explain tumor immune microenvironment heterogeneity of ovarian cancer.

CD47 is a transmembrane protein which plays significant roles in cellular functions, such as proliferation, apoptosis, migration and immune homeostasis (6, 7). CD47 is a ligand for signal regulatory protein alpha (SIRPα) which exists on immune cells, for instance, macrophages and dendritic cells (8). SIRPα binds CD47 to initiate a signaling cascade and finally inhibit phagocytosis (9). Besides, CD47 is a receptor of secreted protein thrombospondin-1 (TSP-1). TSP-1 broadly regulates metabolism in TME and causes resistance to cancer treatment (10). Previous studies used CD47 antibody-B6H12 prevented interaction of TSP-1 and SIRPα with CD47 (10, 11). Blockade of CD47 signaling triggers the immune system, reactivates phagocytosis and promotes tumor eradication (12). Therefore, CD47 could be used as an immunologic protection for targeted therapies (6, 7). However, how CD47 might affect TME heterogeneity of ovarian cancer has not been fully investigated. Therefore, it is urgent to elucidate roles of CD47 in ovarian cancer and to illustrate its immune interactions in ovarian cancer microenvironment.

In this study, we investigated the function and heterogeneity expression pattern of CD47 in different immune cells at single-cell level using CancerSEA and TISCH (13, 14). Several other bioinformatics tools including Oncomine, GEPIA, TIMER were used for CD47 expression analysis. The relationship between CD47 and other parameters, for instance, tumor stage, tumor grade, patient’s race, patient’s age and TP53 mutation status were investigated using Ualcan. KM plotter was used to show how CD47 affect the prognosis of ovarian cancer patients. Moreover, we investigated the correlation of CD47 expression with immune-infiltrating cells in ovarian cancer using TIMER database. To better illustrate CD47 co-expression genes and its regulation networks, Genemania, KEGG and GSEA were further analyzed. This study aims to provide insights into prognosis and immune infiltration-related role of CD47 in ovarian cancer. This study might be a fresh perspective to disclose the reason of heterogeneity of ovarian cancer immune microenvironment.

## Materials and Methods

### Oncomine

Oncomine is a publicly available database (https://www.oncomine.org). It consists online cancer microarray data of 715 datasets, 86,733 cancer and normal tissue samples (15). The Oncomine database was used to illustrate the transcriptional level of CD47 in ovarian cancer. The thresholds were set as follow: *P*-value<1e-4, fold change >2, and gene rank in the top 10%.

### GEPIA

The GEPIA database (http://gepia.cancer-pku.cn/) consists of RNA sequencing expression data of 9,736 tumors and 8,587 normal samples derived from the TCGA and GTEx database (16). CD47 expression level in different cancer types versus normal tissues were processed using GEPIA. *P*<0.05 was considered to be statistically significant.

### CancerSEA

CancerSEA (http://biocc.hrbmu.edu.cn/CancerSEA/home.jsp) is the first dedicated database to analyze distinct functional states of different cancer cells at single-cell level (13). It involves 14 functional states of 41900 cancer single cells in 25 types of different cancers. Cellular functional states include angiogenesis, apoptosis, cell cycle, differentiation, DNA damage, DNA repair, Epithelial-Mesenchymal Transition (EMT), hypoxia, inflammation, invasion, metastasis, proliferation, quiescence and stemness. The CancerSEA database was used to analyze CD47 function in 14 different states using cancer single cell data.

### UALCAN

Ualcan (http://ualcan.path.uab.edu/) is a comprehensive web portal for analyzing cancer OMICS data (17). In this study, expression of CD47 in ovarian cancer was analyzed in Ualcan based on individual cancer stages, tumor grades, patient’s race, patient’s age and TP53 mutation status. The expression level of CD47 was normalized as transcript per million reads. *P*<0.05 was considered statistically significant.

### The Human Protein Atlas

HPA (https://www.proteinatlas.org/) is an open access protein database (18). It is composed of proteomic data based on 26941 antibodies targeting 17165 unique proteins. CD47 protein expression level between ovarian cancer and normal ovary was verified using immunohistochemistry (IHC).

### Kaplan-Meier Plotter

The KM plotter (http://kmplot.com) database evaluates survival of different genes in 21 cancer types including breast (n=6234), ovarian (n=2190), lung (n=3452) and gastric (n=1440) cancer (19). Prognostic values including PFS (progression-free survival) and OS (overall survival) of CD47 were evaluated in ovarian cancers. Four different probes were used to evaluate CD47 related PFS and OS in ovarian cancer. Hazard ratios (HRs) with 95% confidence intervals and logrank *P*-value were determined.

### TISCH

TISCH collected data from Gene Expression Omnibus (GEO) (20) and ArrayExpress (21) to formulate its scRNA-seq atlas (14). TISCH includes 79 databases and 2045746 cells from both tumor patients and healthy donors. The datasets were uniformly processed to enable clarifying components of the TME at both single-cell and annotated cluster levels. In this work, we used datasets derived from TISCH to decipher the TME heterogeneity between primary and metastatic ovarian tumor sites at single cell level.

### GeneMANIA

GeneMANIA (http://genemania.org/) is a flexible website for predicting protein-protein interactions based on gene functions (22). It indexes 2, 813 interactive functional association networks which includes 660, 443, 499 interactions derived from 9 organisms. In this work, we adopted GeneMANIA to construct the network of CD47 interactive proteins based on physical interactions, co-expression, predicted, co-localization, pathway, genetic interactions, and shared protein domains.

### TIMER

TIMER (https://cistrome.shinyapps.io/timer/) is a database for comprehensive analysis of tumor-infiltrating immune cells (23). TIMER database consists of 10897 samples of 32 cancer types from the TCGA database to evaluate immune infiltrates abundance. CD47 expression correlation with six immune infiltrates (B cells, CD4+ T cells, CD8+ T cells, Neutrophils, Macrophages and Dendritic cells) were estimated using TIMER. The gene expression level was assessed using log2 TPM. CD47 was also evaluated with different gene markers of B cell, T cell, CD8+ T cell, Dendritic cell, macrophage type1 cells (M1), macrophage type2 cells (M2), Th1 cell, Th2 cell, regulatory T cells (Treg), monocyte, tumor-associated macrophages (TAM) and T cell exhaustion.

### Pathway Analysis

We used TCGA OV RNA-Seq data to find DEGs between CD47 high and low expression groups with R package DESeq2. There are 376 patients in the TCGA OV RNA-Seq dataset. We used quartile method to divide patients into CD47 high and low group. We sorted descending CD47 TPM expression value in all patients, and chose top 94 patients as CD47 high group, and bottom 96 patients as CD47 low group. The genes with *P* value less than 0.05 and log fold change larger than 1 or lower than -1 were considered significant. They were showed in a volcano plot with R package ggplot2. These different up-expression genes were enrichment in KEGG pathways with KOBAS web tool (http://kobas.cbi.pku.edu.cn/), *P* value less than 0.05 was considered significant in statistic. Besides, we also analyzed pathways in GSEA software about CD47 high expression group.

### Statistical Analysis

Results generated using CancerSEA was displayed with correlation and *P*-value. GEPIA and Ualcan results were calculated using *P*-value. KM plotter and log-rank tests were utilized to represent the survival curves. Results displayed using Oncomine was processed with *P*-values, fold changes and ranks. The correlation of CD47 with different immune infiltration levels was represented using *P*-value. The DEGs analyses were produced using R software (version 3.6.3). *P*<0.05 was considered statistically significant (*P<0.05 **P<0.01, ***P<0.001).

## Results

### CD47 Expression Levels in Different Types of Cancers

**Figure 1:**
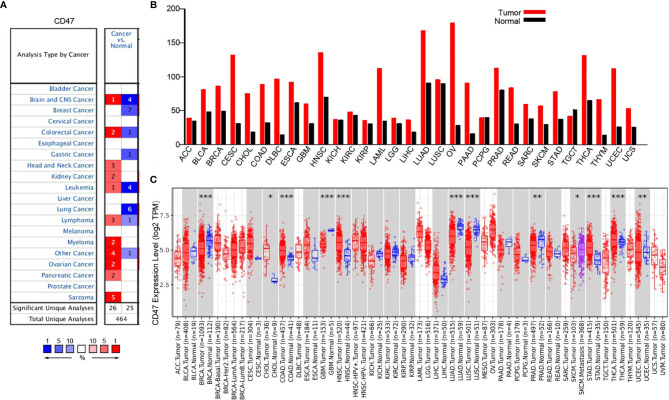
CD47 expression at pan-cancer level. **(A)** CD47 expression level in datasets of distinct cancer types. **(B)** CD47 expression level in different cancers verified using GEPIA database. CD47 was highly expressed in several cancer types, for instance, BLCA, BRCA, CESC, CHOL, COAD and OV. **(C)** CD47 levels in different cancers from TCGA database was represented using TIMER. Significant difference was observed in BRCA, CHOL, COAD, GBM, HNSC, LUAD, LUSC, PRAD, STAD, THCA and UCEC (***p < 0.001, **p < 0.01, *p < 0.05).

Oncomine database was used to probe into CD47 differential expression at pan-cancer level. The results showed high CD47 expression in head and neck cancer, kidney cancer, myeloma, ovarian cancer, pancreatic cancer and sarcoma (**Figure 1A**). Meanwhile, GEPIA database was used to further validate the results. CD47 was highly expressed in bladder urothelial carcinoma (BLCA), BRCA, CESC, cholangiocarcinoma (CHOL), colon adenocarcinoma (COAD) and ovarian cancer (**Figure 1B**). The data was also evaluated using TIMER database, which showed significant difference (*P*<0.05) in BRCA (1093 tumor vs. 112 normal), CHOL, COAD (457 tumor vs. 41 normal), GBM, HNSC, LUAD (515 tumor vs. 59 normal), LUSC, PRAD, STAD, THCA and UCEC (545 tumor vs. 35 normal) (**Figure 1C**). The results showed that CD47 played different roles in distinct types of cancer. Although TIMER only investigated CD47 expression in 303 ovarian tumor samples but not in adjacent normal tissues, CD47 expression level in ovarian cancer was relatively high.

### CD47 Functional States in scRNA-seq Datasets

To explore how CD47 might affect different cancers, we used CancerSEA single-cell database to analyze the correlation of CD47 with 14 distinct functions in 14 types of cancer. As shown in **Supplementary Figure 1**, correlation of CD47 was analyzed using an interactive bubble chart. The upper bar represents the correlation of CD47 with 14 distinct functional states of all single-cell datasets. Expression of CD47 showed positive correlation with angiogenesis, apoptosis, cell cycle, differentiation, EMT, hypoxia, inflammation, metastasis, proliferation and quiescence. The data showed that CD47 might play a tumor activator role in several types of cancer.

### Functional Characteristics of CD47 in Different Cancers

**Figure 2:**
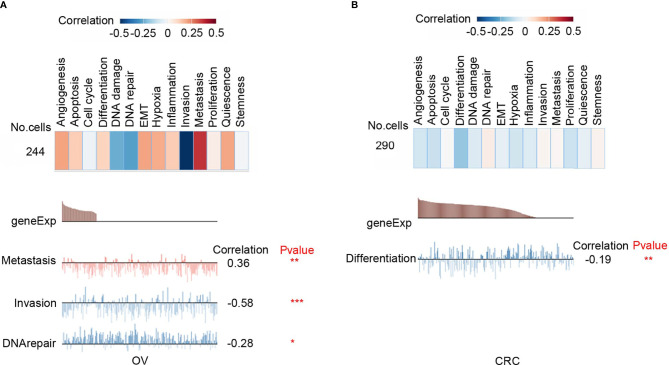
Functional relevance of CD47 in cancers. **(A)** Functional relevance of CD47 in OV. **(B)** Functional relevance of CD47 in CRC. (***p < 0.001, **p < 0.01, *p < 0.05).

Functional relevance analysis showed that CD47 expression was positively correlated with metastasis in ovarian carcinoma (**Figure 2A**); with DNA repair in renal cell carcinoma (RCC) (**Supplementary Figure 2A**); with inflammation, cell cycle, DNA repair, and proliferation in breast cancer (BRCA) (**Supplementary Figure 2B**); with invasion, quiescence, proliferation, and angiogenesis in prostate cancer (PC) (**Supplementary Figure 2C**); with metastasis, invasion and EMT in head and neck cancer (**Supplementary Figure 2D**); with invasion, EMT, hypoxia and metastasis in glioblastoma (GBM) (**Supplementary Figure 2E**); with metastasis, cell cycle, proliferation and EMT in glioma (**Supplementary Figure 2F**); with inflammation, differentiation, quiescence and hypoxia in astrocytoma (AST) (**Supplementary Figure 2G**); with metastasis, EMT, inflammation and apoptosis in high-grade glioma (HGG) (**Supplementary Figure 2H**); with stemness, inflammation, differentiation and metastasis in Oligodendroglioma (ODG) (**Supplementary Figure 2I**); with no significant correlation in lung adenocarcinoma (LUAD) (**Supplementary Figure 2J**); with quiescence, inflammation, proliferation and differentiation in non-small cell lung cancer (NSCLC) (**Supplementary Figure 2K**); with metastasis, quiescence, apoptosis and inflammation in melanoma (MEL) (**Supplementary Figure 2L**).

However, CD47 is negatively correlated with invasion (*R*=-0.58; *P*<0.001) and DNA repair (*R*=-0.28; *P*<0.05) in ovarian cancer (**Figure 2A**); with differentiation (*R*=-0.19; *P*<0.01) in colorectal cancer (CRC) (**Figure 2B**). These data indicated that CD47 might play different functional roles in ovarian cancer and CRC. Therefore, in this study, we aimed to clarify functional role of CD47 in ovarian cancer.

### CD47 Expression Level in Ovarian Cancer Patients

**Figure 3:**
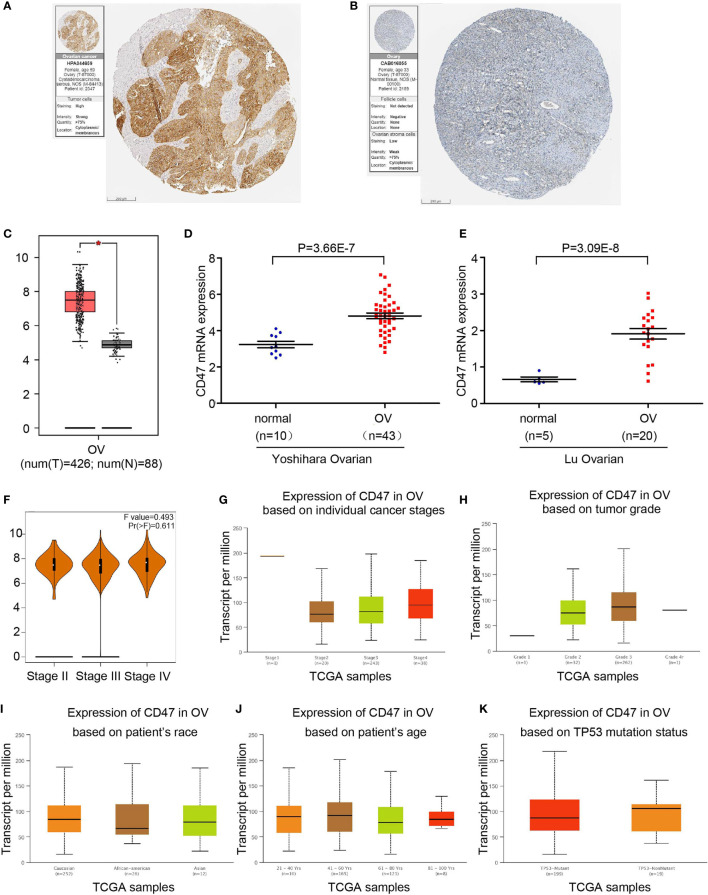
CD47 transcription levels in ovarian cancer. **(A)** Protein levels of CD47 in ovarian cancer tissue. **(B)** Protein levels of CD47 in normal ovary. **(C)** Boxplot shows expression of CD47 in ovarian cancer using GEPIA. **(D)** Box plot shows CD47 mRNA levels in Yoshihara ovarian dataset. **(E)** Box plot shows CD47 mRNA levels in Lu ovarian dataset. **(F)** CD47 expression level has no correlation among different stages in OV. **(G)** Expression of CD47 based on individual cancer stages using Ualcan database. **(H)** Expression of CD47 based on tumor grades. **(I)** Expression of CD47 based on patient’s race. **(J)** Expression of CD47 based on patient’s age. **(K)** Expression of CD47 based on TP53 mutation status. For all the analysis, p<0.05 was considered statistically significant.

First, we used the HPA database to confirm CD47 expression in ovarian cancer and normal ovary tissue. CD47 expression level in ovarian cancer was significantly higher than that of normal ovary tissue (**Figures 3A, B**). CD47 expression level was notably higher in ovarian cancer compared to normal ovarian tissues (*P*<0.05; num (T)=426; num (N)=88) (**Figure 3C**). Two independent datasets derived from oncomine showed that CD47 was highly expressed in ovarian cancer in Yoshihara Ovarian (*P*=3.66e-7) and Lu Ovarian (*P*=3.09e-8) (**Figures 3D, E**). GEPIA and Oncomine results were consistent with the HPA database.

We next investigated CD47 expression based on cancer stages using GEPIA database. Expression level of CD47 in ovarian cancer showed no significant difference among different stages (**Figure 3F**). Ualcan database demonstrated no correlation of CD47 expression with cancer stages, tumor grade, patient’s race, patients’ age or TP53 mutation status (**Figures 3G–K**). These results suggest that CD47 may serve as a diagnostic tumor marker in ovarian cancer regardless of different pathological parameters.

### Prognostic Value of CD47 in Ovarian Cancers

**Figure 4:**
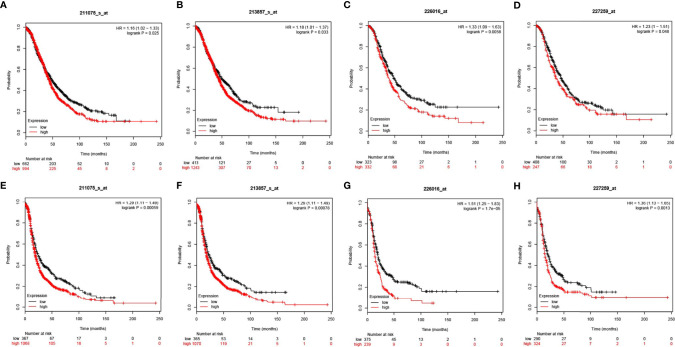
Prognostic values of CD47 in OV using different probes. CD47 OS in OV using probes of **(A)** 211075_s_at, **(B)** 213857_s_at, **(C)** 226016_at, **(D)** 227259_at. CD47 PFS in OV using probes of **(E)** 211075_s_at, **(F)** 213857_s_at, **(G)** 226016_at, **(H)** 227259_at.

The KM plotter was used as an indicator for evaluating prognostic value of CD47 expression based on Affymetrix microarrays. CD47 mRNA level correlated with overall survival (OS) and progression-free survival (PFS) in ovarian cancer patients. We found that CD47 mRNA expression level was positively correlated with worse OS among all ovarian cancer patients based on four different arrays (HR=1.16 (1.02-1.33), logrank P=0.025 for 211075_s_at; HR=1.18 (1.01-1.37), logrank P=0.033 for 213857_s_at; HR=1.33 (1.09-1.63), logrank P=0.0058 for 226016_at and HR = 1.23 (1-1.51), logrank P = 0.048 for 227259_at (**Figures 4A–D**). We found CD47 mRNA expression level reversely correlated with PFS of ovarian cancer patients with HR=1.29 (1.11-1.49), logrank P=0.00059 for 211075_s_at; HR=1.29 (1.11-1.49), logrank P=0.00078 for 213857_s_at; HR=1.51 (1.25-1.83), logrank P=1.7e-05 for 226016_at and HR=1.36 (1.13-1.65), logrank P=0.0013 for 227259_at (**Figures 4E–H**). The data suggested CD47 to be a potential biomarker for predicting ovarian cancer prognosis.

### Correlation Between CD47 and the Tumor Immune Microenvironment Heterogeneity

**Figure 5:**
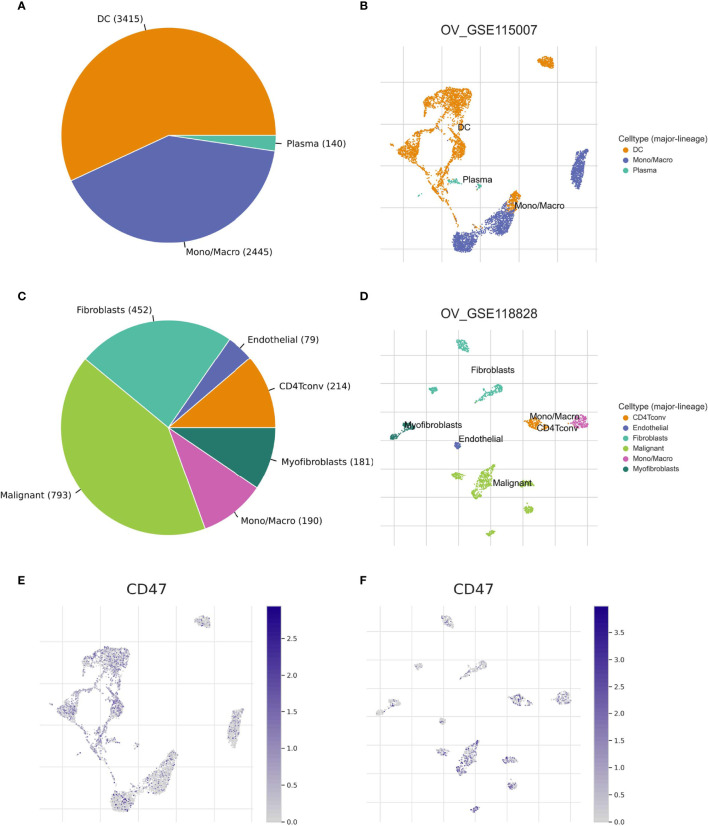
CD47-related cell type distribution using scRNA seq database. **(A–D)** The cell types and their distribution in OV_GSE115007 and OV_GSE118828 datasets. **(E, F)** Distribution of CD47 in different cells in OV_GSE115007 and OV_GSE118828 datasets.

We used two datasets (OV_GSE115007 and OV_GSE118828) of the TISCH database to evaluate CD47 expression in TME-related immune cells. As to different immune cell types, for instance, plasma cells, dendritic cells and Mono/Macro cells of OV_GSE115007, myofibroblasts, fibroblasts and endothelial cells of OV_GSE118828, the CD47 expression level was relatively low compared to expression level in malignant cells of OV_GSE118828 dataset (**Supplementary Figure 3A**). In OV_GSE118828 dataset, CD47 expression level remains the highest in malignant cells, suggesting high CD47 expression in malignant ovarian cancer cells (**Supplementary Figure 3A**). As to other components of the TME, relatively higher CD47 expression level was observed in CD4 T conventional cells and mono/macro cells of OV_GSE118828 dataset. Violin plot showed the same trend of CD47 expression in the ovarian cancer cell microenvironment (**Supplementary Figure 3B**). In OV_GSE115007, only three cell types were found, including plasma cells, dendritic cells and Mono/Macro cells, while DC cells exhibited the most abundant cell counts (n=3415) (**Figures 5A, B**). And all of the three cell types exhibited CD47 expression at a lower level compared to malignant cells in OV_GSE118828 dataset. While in OV_GSE118828 dataset, six cell types were found, with the highest number of 793 to malignant cells (**Figures 5C, D**). Because it was reported that the OV_GSE115007 contains only tumor ascites from primary ovarian cancer patients and OV_GSE118828 dataset contains both primary and metastatic tumor sites (24, 25), we got the fact that the cell components and distribution in primary ovarian cancer ascites and metastatic ovarian cancers are quite distinct. **Figures 5E, F** represented the distribution of various immune cells related to **Figures 5B, D**. These results suggest that CD47 expression level was quite different in distinct cell types with the highest in malignant ovarian cancer cells and the cell components of primary and metastatic patients are different, which might be the source of ovarian cancer microenvironment heterogeneity.

### CD47 Expression Is Correlated With Immune Infiltration in Ovarian Cancer

**Figure 6:**
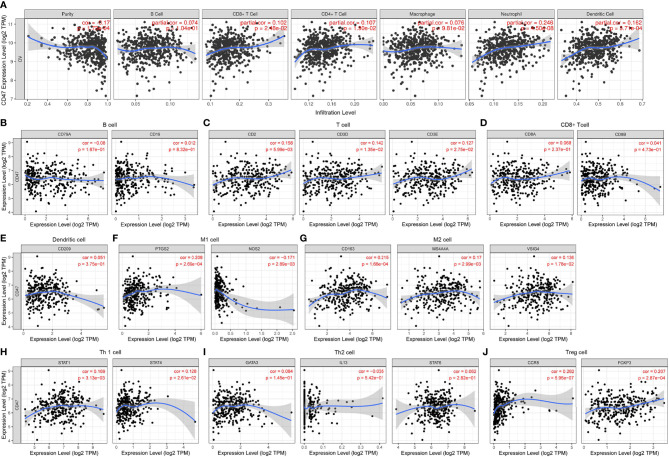
Expression of CD47 is related to a panel of gene markers of immune cells. **(A)** The relation of CD47 expression and immune cell infiltration in OV using TIMER. Relation of CD47 is analyzed with gene markers of **(B)** B cell, **(C)** T cell, **(D)** CD8+ T cell, **(E)** Dendritic cell, **(F)** M1 cell, **(G)** M2 cell, **(H)** Th1 cell, **(I)** Th2 cell, **(J)** Treg cell.

To further clarify ovarian cancer microenvironment and how CD47 might affect ovarian cancer heterogeneity and prognosis, we investigated whether CD47 was related to immune infiltration. TIMER database was used to estimate immune infiltration levels in ovarian cancer. CD47 expression was positively correlated with CD8+ T cell infiltration (r=0.102, p=2.48e-02), CD4+ T cell (r=0.107, p=1.90e-02), Neutrophil (r=0.246, p=4.50e-08) and dendritic cell (r=0.162, p=3.71e-04), negatively correlated with tumor purity (r=-0.17, p=1.73e-04). No correlation was observed with B cell (r=0.074, p=1.04e-01) and macrophage (r=0.076, p=9.81e-02) (**Figure 6A**). In addition, we evaluated CD47 expression with other immune cells markers. The results showed that CD47 was not correlated with B cell (**Figure 6B**), CD8+ T cell (**Figure 6D**), dendritic cell (**Figure 6E**) and Th2 cell (**Figure 6I**), but correlated with other immune cells of T cell (**Figure 6C**), M1 cell (**Figure 6F**), M2 cell (**Figure 6G**), Th1 cell (**Figure 6H**), Treg cell (**Figure 6J**).

To illustrate whether CD47 is correlated with other immune infiltrating cells, we also analyzed its correlation with monocyte and TAM cells. CD47 expression was positively correlated with monocyte and TAM infiltration in ovarian cancer (**Supplementary Figures 4A, B**). We then evaluated correlation of T cell exhaustion biomarkers PD1 (PDCD1), CTLA4, LAG3 and HAVCR2 with the expression of CD47 in ovarian cancer (**Supplementary Figure 4C**). The results showed that CD47 was positively correlated with T cell exhaustion. In summary, the above results suggested that CD47 expression might affect the TME through regulating immune cell infiltration.

### Interaction Networks Between CD47 and Its Interactive Genes

We analyzed the interactive networks of CD47 with its interactive genes using the GeneMANIA database (**Supplementary Figure 5**). The circle represented CD47 gene located at center of the interaction network. CD47 gene was surrounded by 20 circles representing genes showing close relation based on physical interactions, co-expression, predicted, co-localization, pathway, genetic interactions and shared protein domains. The top five genes included the signal regulatory protein gamma (SIRPG), SIRPα, src kinase associated phosphoprotein2 (SKAP2), protein tyrosine kinase 2 beta (PTK2B) and the thrombosondin1 (THBS1) gene. The five genes displayed greatest correlation with the CD47 gene. Further analysis showed that these 20 closely correlated genes were related with functions of cell adhesion mediator activity, leukocyte migration, cell-substrate adhesion, regulation of reactive oxygen species metabolic process, leukocyte cell-cell adhesion, cellular extravasation and sulfur compound binding.

### Immune-Related Pathways Regulated by CD47 in Ovarian Cancer

**Figure 7:**
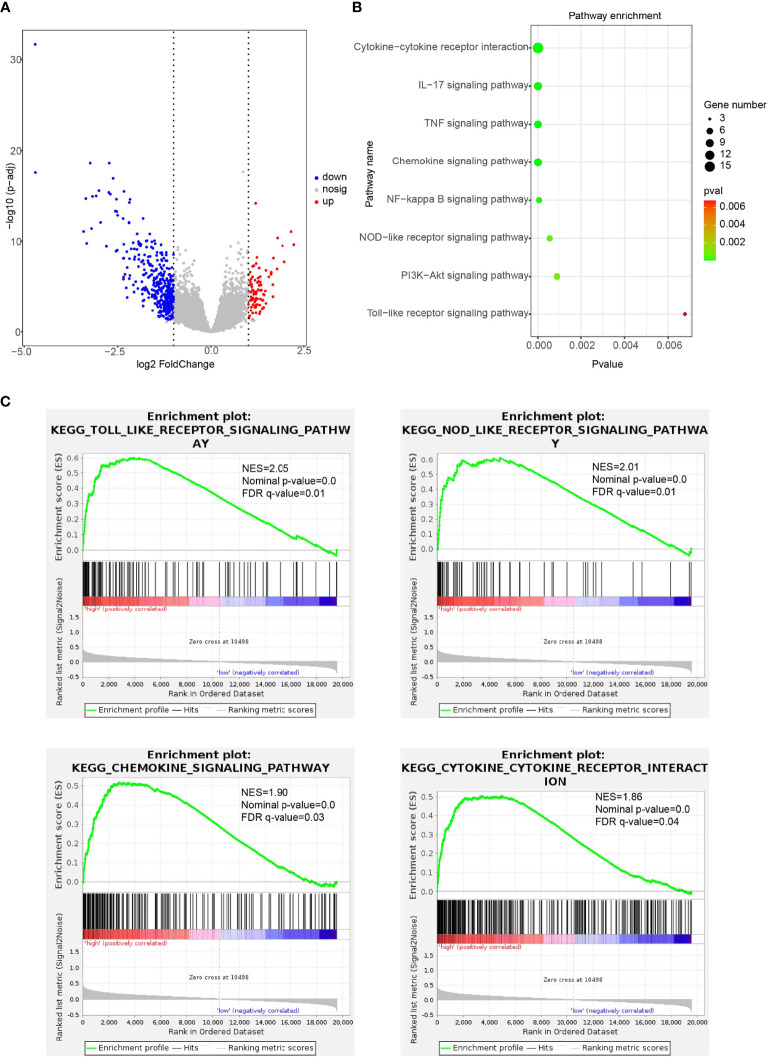
Immune-related pathways regulated by CD47 in ovarian cancer detected using KEGG and GSEA analysis. **(A)** CD47 high expression associated DEGs in ovarian cancer. **(B)** Biological processes related to CD47 high expression in ovarian cancer. **(C)** GSEA analysis revealed CD47 expression involved in the process of different pathways.

To explore the association of CD47 high expression with immune regulation, we performed KEGG and GSEA analysis. First, we found CD47 high expression associated DEGs in ovarian cancer (**Figure 7A**). Then, KEGG pathway enrichment was performed to explore the biological processes related to CD47 high expression in ovarian cancer. Biological processes were mainly involved in cytokine-cytokine receptor interaction, IL-17 signaling pathway, TNF signaling pathway, chemokine signaling pathway, NF-kappa B signaling pathway, NOD-like receptor signaling pathway, PI3K-Akt signaling pathway and Toll-like receptor signaling pathway (**Figure 7B**). According to GSEA analysis, high CD47 expression was mainly involved in the process of toll-like receptor signaling pathway (NES=2.05, p-value=0.0), NOD-like receptor signaling pathway (NES=2.01, p-value=0.0), chemokine-signaling pathway (NES=1.90, p-value=0.0) and cytokine-cytokine receptor interaction pathways (NES=1.86, p-value=0.0) (**Figure 7C**). Accordingly, these above data suggest that CD47 high expression is closely linked to ovarian cancer cell immune cell infiltration and thus might affect TME and induce ovarian cancer heterogeneity.

## Discussion

CD47 was reported to be involved in various biological processes, including neutrophil migration, macrophage phagocytosis, immune system homeostasis and other immune-related cellular processes (26, 27). Increased CD47 impression has been reported in ovarian cancer (28, 29). Blockade of CD47 signaling reactivates phagocytosis and promotes tumor eradication (6, 7). However, how CD47 might be correlated with ovarian cancer immune microenvironment and lead to tumor heterogeneity has not been fully studied.

Firstly, we used oncomine, GEPIA and TIMER databases to investigate CD47 expression at pan-cancer level. We found CD47 highly expressed in many types of cancers, including ovarian cancer (**Figure 1**). To illustrate functional roles of CD47, scRNA-seq database-CancerSEA was further implemented. We found CD47 positively connected to various functions in most cancer types. However, in ovarian cancer, CD47 was positively correlated to metastasis, negatively correlated to invasion and DNA repair (**Figure 2**). The distinct functional roles of CD47 in ovarian cancer might be due to inherent heterogeneity in ovarian cancer or the small amount of single cells collected using scRNA-seq data.

To further explain correlation of CD47 with prognosis of ovarian cancer, we used several bioinformatics database including HPA, oncomine, GEPIA, Ualcan and KM plotter. The results showed that CD47 was highly expressed in ovarian cancer which was consistent with previous reports (12, 29). HPA database confirmed the result using immunohistochemistry. Ualcan concluded that CD47 expression level was independent of patients’ stages, grades, age, race, or TP53 mutation status. Higher CD47 expression was connected to poorer OS and PFS in ovarian cancer. Together we found strong evidence that CD47 might be used as a prognostic biomarker in ovarian cancer patients.

It is well known that the TME of ovarian cancer is highly heterogeneous (30, 31). To clarify how CD47 might affect TME, we used TISCH single cell database. We observed different immune cell distribution based on primary and primary to metastatic ovarian tumor sites. In OV_GSE115007 dataset, tumor ascites from primary ovarian cancer patients exhibited relatively low CD47 expression levels in plasma cells, dendritic cells and Mono/Macro cells compared to expression level in malignant cells of OV_GSE118828 dataset (**Supplementary Figure 3A**). In contrast, in primary to metastatic ovarian tumor sites, OV_GSE118828 exhibited more cell types, including CD4 T conventional cells, myofibroblasts, fibroblasts and endothelial cells. Higher CD47 expression was observed in mono/macro cells in OV_GSE118828 dataset compared to that of OV_GSE115007 dataset. And that CD47 expression was higher in malignant ovarian cells in OV_GSE118828 dataset. Therefore, the TME in primary and metastatic ovarian cancer might be different, leading to heterogeneity of ovarian cancer.

It was reported that tumor immune cell infiltration might be related to prognosis of cancers (32, 33). Presence of CD8+ T cells in ovarian cancer is associated with prolonged survival (34, 35). Zhou et al. reported the availability of considering immune cells in diagnosis and treatment of colon cancer (36). Burugu et al. suggested that both density and distribution of immune cells could affect prognosis of breast cancer (37). These researches highlight the role of immune infiltration in prognosis of cancers. However, there is limited research probing into the role of CD47 regarding immune infiltration in ovarian cancer. In our study, we used TIMER to investigate different tumor immune cell infiltration in ovarian cancer. The results showed that M2 and Tregs were increasingly infiltrated in high CD47 ovarian cancer microenvironment (**Figure 6**). M2 and Tregs were capable of making immunologic barriers against antitumor immune responses, indicating immune escape in CD47 high expression ovarian cancer (38).

Correlation between CD47 and biomarkers of PD1, CTLA4, LAG3, HAVCR2 showed that CD47 expression level was positively correlated with T cell exhaustion (**Supplementary Figure 4**). Because T cell exhaustion leads to immune escape, therefore, cancer cells get out of control from the immune system (39). In numerous models and clinical trials, CD47 has been blocked to promote phagocytosis. Blockade of CD47 reduced tumor burden both *in vitro* and *in vivo* (40, 41). Therefore, cancer therapies targeting CD47 might be used to treat ovarian cancer.

We next explored the gene networks correlated to CD47. We showed that CD47 expression interacted with SIRPG, SIRPA, SKAP2, PTK2B, TSP-1 and 15 other proteins. These proteins closely related with CD47 were mainly involved with functions of cell adhesion mediator activity, leukocyte migration and leukocyte cell-cell adhesion. These suggested that CD47 might affect ovarian cancer tumorigenesis and prognosis through regulating TME.

KEGG and GSEA analysis indicated that CD47 high expression in ovarian cancer was mainly involved in toll-like receptor signaling pathway, NOD-like receptor signaling pathway, chemokine-signaling pathway and cytokine-cytokine receptor interaction pathways. The results suggested that CD47 might be crucial in regulating tumorigenesis and progression. For instance, the Toll-like receptors (TLRs) are expressed in immune cells as well as tumor cells. Abnormal activation of TLRs can induce immune reaction in tumor cells and thereby modulate the TME (42). NOD-like receptors (NLRs) are reported to be capable of activating innate immune responses (43). NLRs play crucial roles in immune functions, metastasis and tumorigenesis (44, 45). As to chemokine-signaling pathway, chemokines are known to be involved with immune evasion and inflammatory reactions of many kinds of cancers. Chemokine-receptor interactions are reported to be connected to TME modification of cancers (46). Cytokine-cytokine receptor interaction is closely connected with immune reactions. And cytokines are intricately linked with prognosis of patients in various cancers (45, 47). The roles of CD47 might help clarify the mechanism and detailed processes of these pathways.

Accordingly, these above data suggest that CD47 high expression is closely linked to ovarian cancer cell immune infiltration and thus affect TME and might induce ovarian cancer heterogeneity. These findings underscore CD47 expression with different types of immune cell infiltration and T cell exhaustion. Therefore, CD47 high expression might contribute to immune escape, leading to worse prognosis of ovarian cancer.

## Conclusions

In summary, our study showed that increased levels of CD47 could impact the ovarian cancer TME, indicating that CD47 might be used as a potential predictor of ovarian cancer heterogeneity. Moreover, our results demonstrated that CD47 expression level was correlated with ovarian cancer immune infiltration level. Therefore, CD47 might play vital roles in affecting immune infiltration in ovarian cancer microenvironment and be used as a potential target in reversing immune escape and provide insights into understanding the function of CD47 in ovarian cancer prognosis and tumor immunology.

## Data Availability Statement

The datasets presented in this study can be found in online repositories. The names of the repository/repositories and accession number(s) can be found in the article/**Supplementary Material**.

## Author Contributions

LY and YD conceived the idea for the paper and were major contributors in writing the manuscript. TW and TD participated in the design. HH and JL revised the manuscript. LY and JL provided fund. All authors contributed to the article and approved the submitted version.

## Funding

This work was supported by the National Natural Science Foundation of China (No. 81772782 and No. 81972443), Key scientific research platforms and research projects of universities in Guangdong Province (2018KQNCX204), the Guangdong basic and applied basic research fund project-regional joint fund (2019A1515110046), and the General guidance project of Health Science and technology in Guangzhou (20201A011074).

## Conflict of Interest

The authors declare that the research was conducted in the absence of any commercial or financial relationships that could be construed as a potential conflict of interest.

## Publisher’s Note

All claims expressed in this article are solely those of the authors and do not necessarily represent those of their affiliated organizations, or those of the publisher, the editors and the reviewers. Any product that may be evaluated in this article, or claim that may be made by its manufacturer, is not guaranteed or endorsed by the publisher.

## Supplementary Material

Supplementary Figure 1 Relevance of CD47 among 14 functional states in distinct cancers. The upper bar represents the correlation of CD47 with 14 distinct functional states of all single-cell datasets. Red and blue represent positive and negative correlation respectively.

Supplementary Figure 2 Functional correlation of CD47 in distinct cancers. CD47 shows distinct functional relevance in twelve different cancers. (***p < 0.001, **p < 0.01, *p < 0.05, ns means p > 0.05).

Supplementary Figure 3 Correlation between CD47 and the tumor immune microenvironment heterogeneity using TISCH. Average expression of CD47 in different cell types **(A)**. Distribution of CD47 expression in different cell types using violin plot **(B)**.

Supplementary Figure 4 Expression of CD47 is related to immune infiltration of TME in ovarian cancer. Immune infiltration levels represented using gene markers of **(A)** monocyte, **(B)** TAM and **(C)** T cell exhaustion.

Supplementary Figure 5 Interaction networks between CD47 and its interactive genes using GeneMANIA.

## Abbreviations

SIRPα, signal regulatory protein alpha; cancer single-cell state atlas (CancerSea); GEPIA, Gene Expression Profiling Interaction Analysis; TIMER, Tumor Immune Estimation Resource; KM plotter, Kaplan-Meier plotter; EMT, Epithelial-Mesenchymal Transition; HPA, The Human Protein Atlas; IHC, immunohistochemistry; PFS, progression-free survival; OS, overall survival; HRs, Hazard ratios; M1 cells, macrophage type1 cells; M2 cells, macrophage type2 cells; Treg, regulatory T cells; TAMs, Tumor-associated macrophages.
