# Supplementary figures and images for: Significance of CD47 and Its Association With Tumor Immune Microenvironment Heterogeneity in Ovarian Cancer
Source: Front Immunol. 2021 Dec 13;12:768115. doi: 10.3389/fimmu.2021.768115 (PMC8710451; doi:10.3389/fimmu.2021.768115)

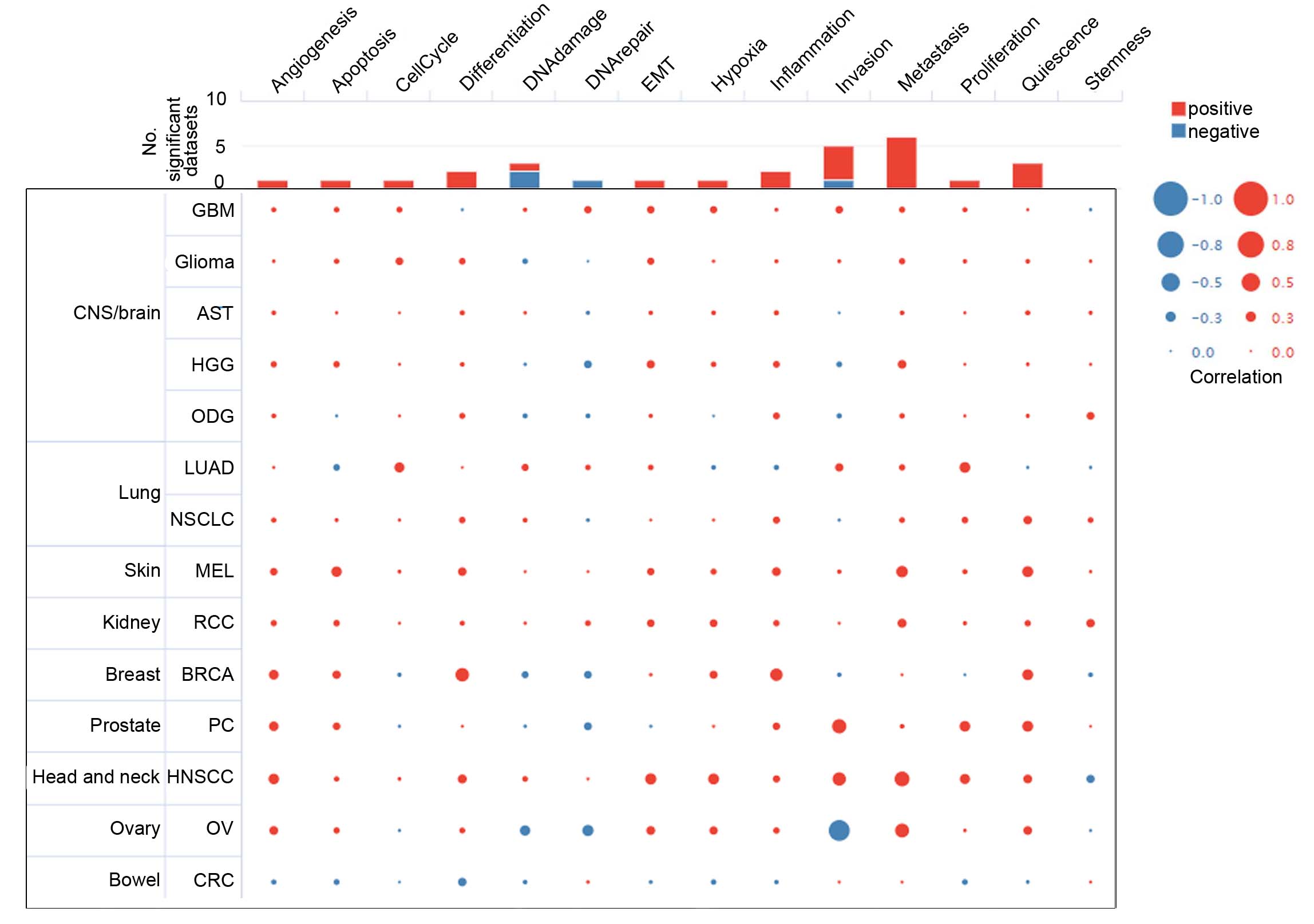

Supplement: Supplementary Figure 1 — Relevance of CD47 among 14 functional states in distinct cancers. The upper bar represents the correlation of CD47 with 14 distinct functional states of all single-cell datasets. Red and blue represent positive and negative correlation respectively. [file Image_1.jpeg]

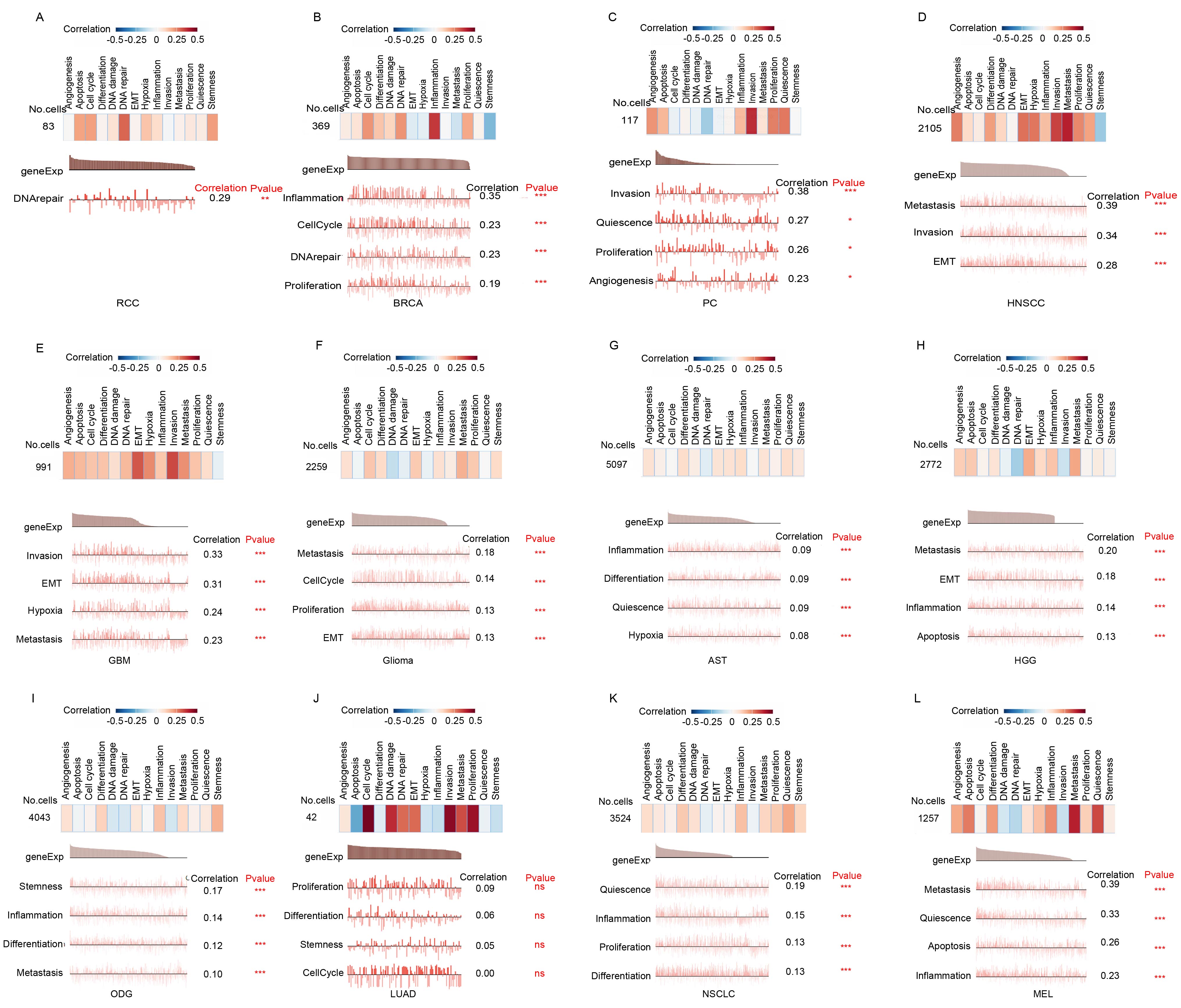

Supplement: Supplementary Figure 2 — Functional correlation of CD47 in distinct cancers. CD47 shows distinct functional relevance in twelve different cancers. (***p < 0.001, **p < 0.01, *p < 0.05, ns means p > 0.05). [file Image_2.jpeg]

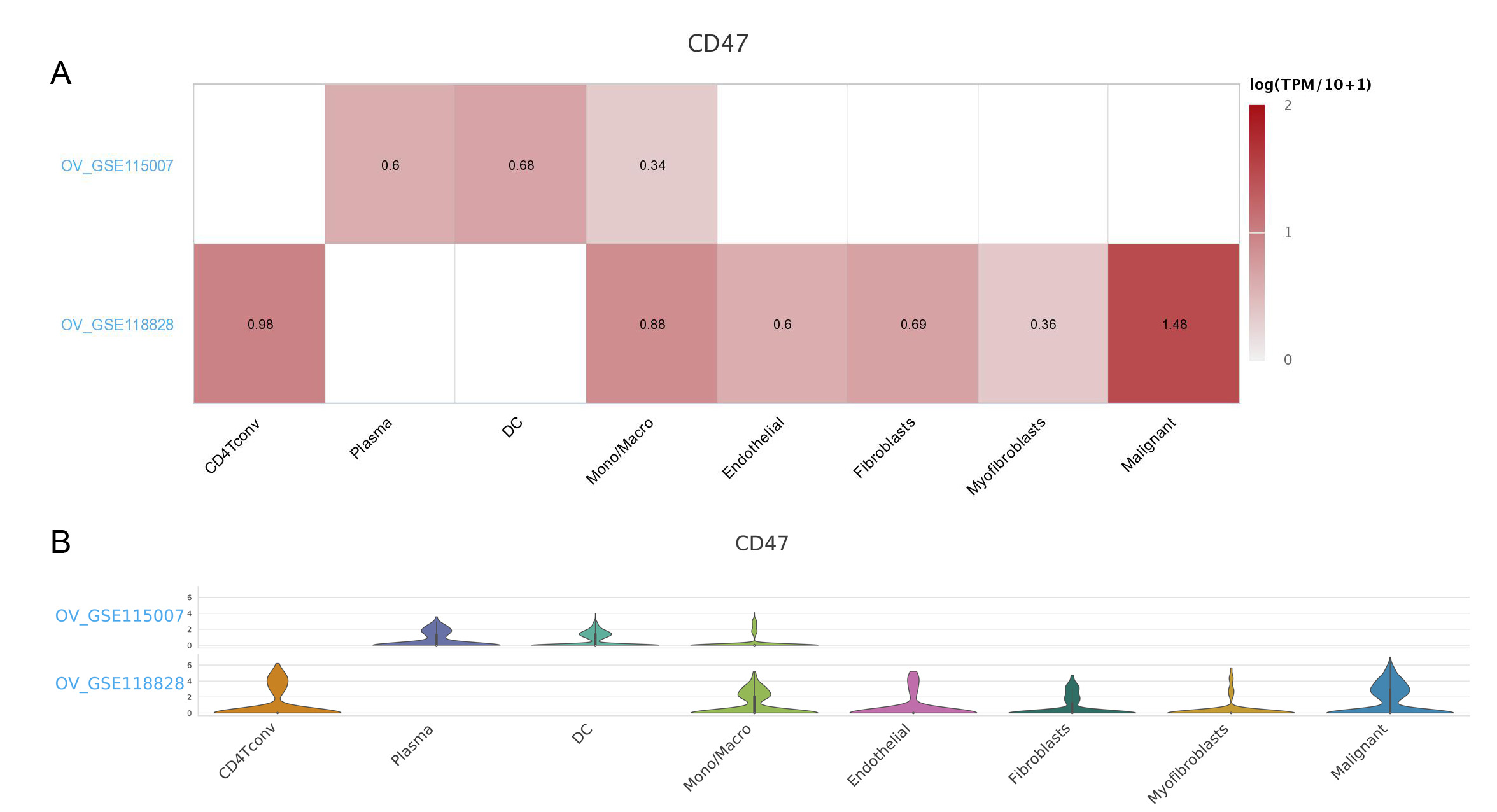

Supplement: Supplementary Figure 3 — Correlation between CD47 and the tumor immune microenvironment heterogeneity using TISCH. Average expression of CD47 in different cell types (A). Distribution of CD47 expression in different cell types using violin plot (B). [file Image_3.jpeg]

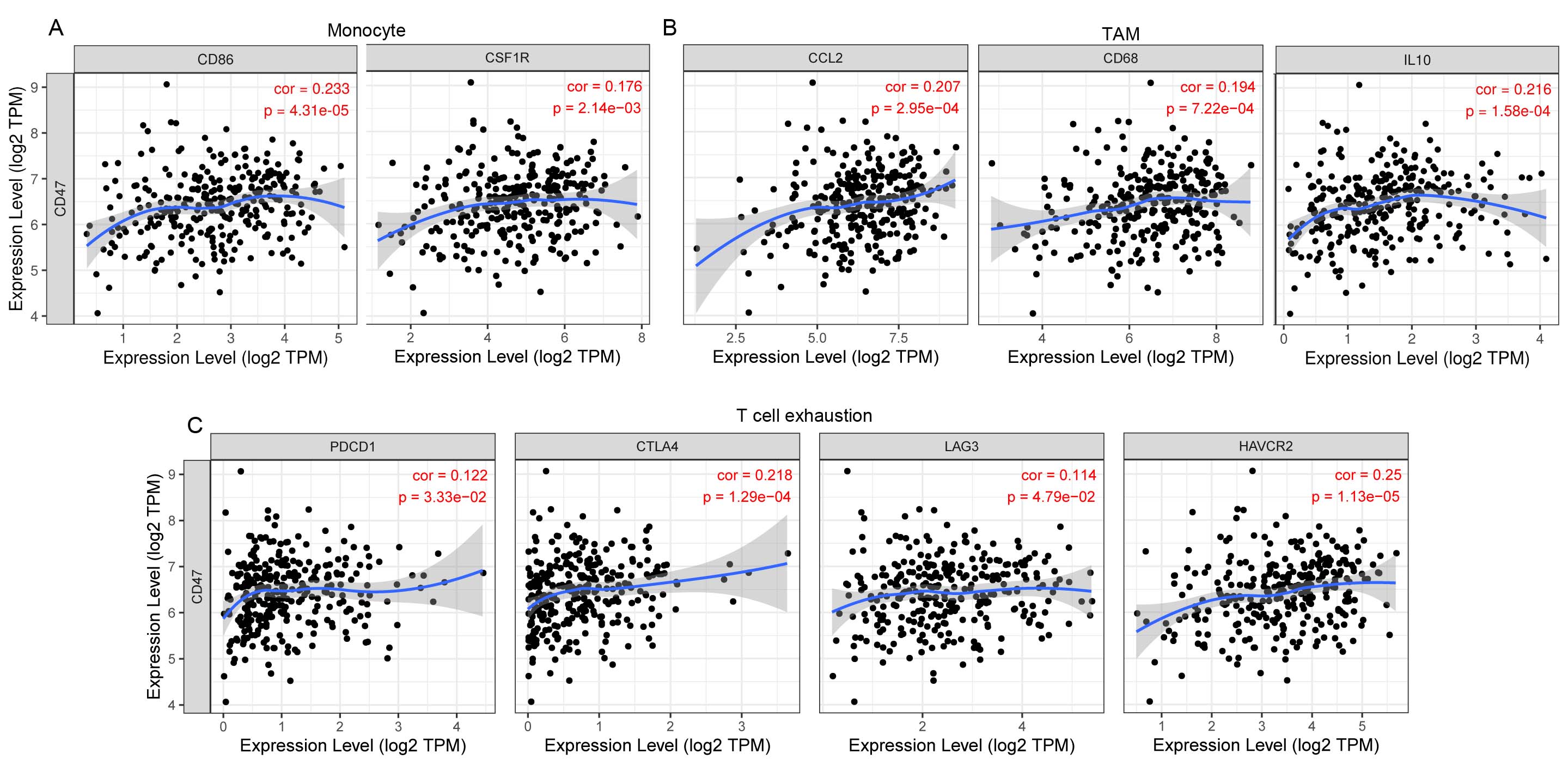

Supplement: Supplementary Figure 4 — Expression of CD47 is related to immune infiltration of TME in ovarian cancer. Immune infiltration levels represented using gene markers of (A) monocyte, (B) TAM and (C) T cell exhaustion. [file Image_4.jpeg]

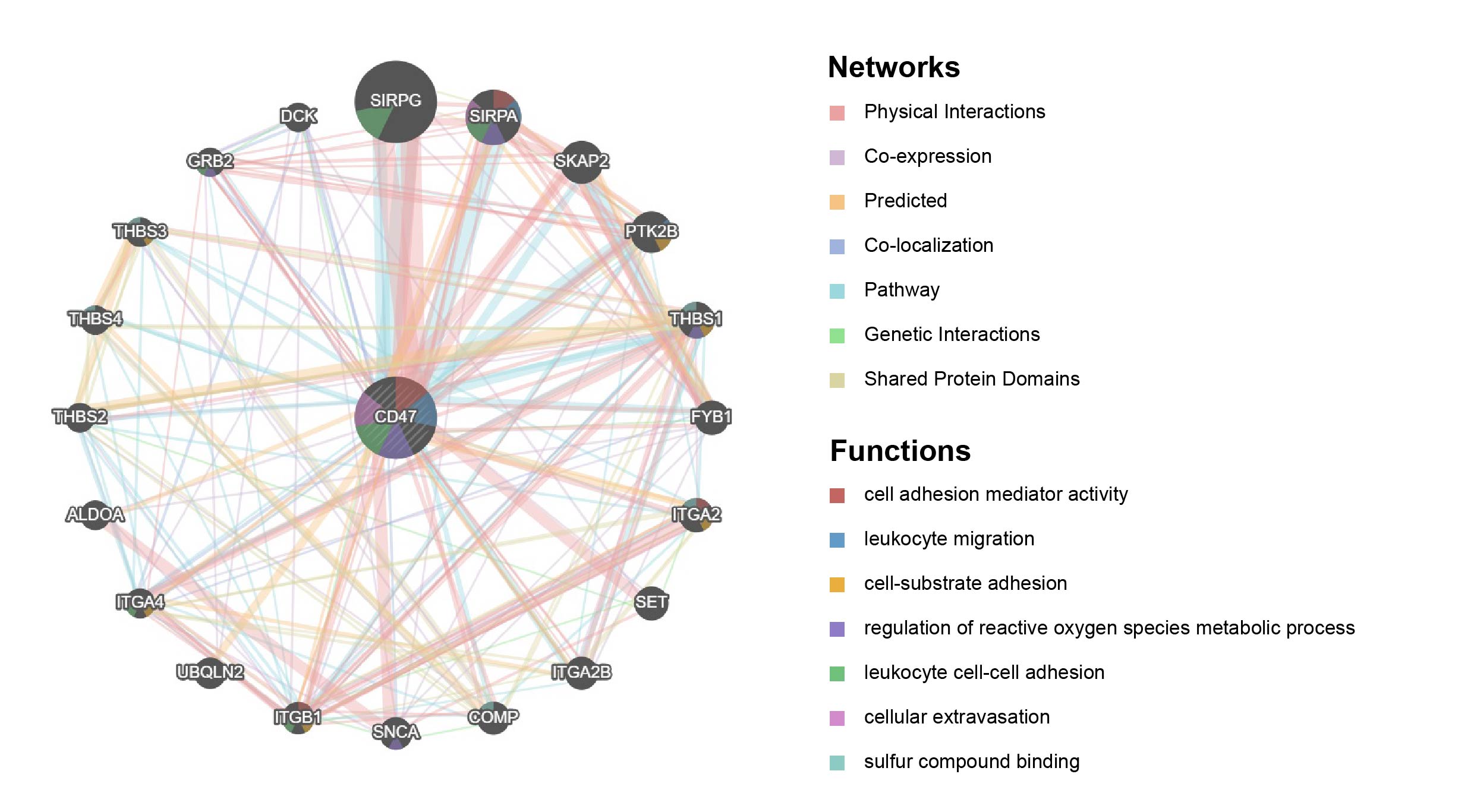

Supplement: Supplementary Figure 5 — Interaction networks between CD47 and its interactive genes using GeneMANIA. [file Image_5.jpeg]
